# Adherence of Obese Patients from Poland and Germany and Its Impact on the Effectiveness of Morbid Obesity Treatment

**DOI:** 10.3390/nu14183880

**Published:** 2022-09-19

**Authors:** Karolina Hoffmann, Dorota Kopciuch, Michał Michalak, Wiesław Bryl, Krzysztof Kus, Kinga Marzec, Jonas Raakow, Matthias Pross, Rafael Berghaus, Elżbieta Nowakowska, Magdalena Kostrzewska, Tomasz Zaprutko, Piotr Ratajczak, Anna Paczkowska

**Affiliations:** 1 Department of Internal Diseases, Metabolic Disorders and Arterial Hypertension, Poznan University of Medical Sciences, 60-572 Poznań, Poland; 2 Department of Pharmacoeconomics and Social Pharmacy, Poznan University of Medical Sciences, 60-806 Poznań, Poland; 3 Department of Computer Science and Statistics, Poznan University of Medical Sciences, 60-806 Poznań, Poland; 4 Department of Surgery, Campus Charité Mitte and Campus Virchow Klinikum, Charité—Universitätsmedizin, 10117 Berlin, Germany; 5 Department of Surgery, DRK Kliniken Berlin, Köpenick, 12559 Berlin, Germany; 6 Department of Pharmacology and Toxicology, Institute of Health Sciences, Collegium Medicum, University of Zielona Góra, 65-516 Zielona Góra, Poland; 7 Department of Pulmonology, Allergology and Pulmonological Oncology, Poznan University of Medical Sciences, 60-572 Poznań, Poland

**Keywords:** obesity, morbid obesity, adherence, compliance

## Abstract

This study aimed to investigate and compare the adherence of patients treated for morbid obesity living in Poland and Germany. **Methods:** A cross-sectional international multicenter survey design was adopted. The study involved 564 adult subjects treated for morbid obesity at selected healthcare facilities in Germany (210 participants) and Poland (354 participants). A validated, custom-made questionnaire based on the literature related to this issue was used. **Results:** The degree of adherence was higher, but not statistically significant, among Polish patients (83.82% vs. 78.33%, *p* = 0.26140). Patient adherence was associated with gender, age, level of education, duration of obesity, number of health professionals involved in obesity treatment, and type of obesity treatment (*p* < 0.05). A positive correlation was observed in the case of age, level of education, and a growing number of health professionals involved in obesity treatment, whereas a negative correlation was observed in the case of the duration of obesity. Patients who underwent bariatric surgery significantly more often followed medical recommendations regarding lifestyle changes, compared to obese participants treated only conservatively. Adherence in the field of obesity treatment significantly increases the percentage of total weight loss and excess weight loss due to applied obesity treatment among both Polish and German groups (*p* < 0.001). Both the percentage of total weight loss and that of excess weight loss were significantly higher in the group of adherent patients compared to the nonadherent patients (*p* < 0.00001). The levels of perceived anxiety, stress, and depression were significantly higher in nonadherent patients in both countries. **Conclusions:** These findings confirm the role of adherence in the effective and satisfactory treatment of morbid obesity. There is a great need to improve patient adherence to overcome the consequences of the obesity pandemic.

## 1. Introduction

The global prevalence of obesity tripled and the prevalence of overweight almost doubled between 1975 and 2016, with 13.2% of adults classified as having obesity, and 39.1% as overweight in 2016 [1]. In the United States of America, obesity grade 3, called morbid (or “severe”) obesity, defined as having a body mass index (BMI) above 40 kg/m^2^, affects 9.2% of adults, more often females (11.5%) than males (6.9%) [2]. The prevalence of severe obesity was highest among people aged 40–59 (11.5%), followed by individuals aged 20–39 (9.1%) and adults aged 60 and over (5.8%) [2]. It is also estimated that the global prevalence of morbid obesity will surpass the prevalence of underweight by 2025 [1]. In the European Union (EU), 30–70% of adults are overweight and 10–30% of adults are obese [3]. In Poland, according to the data from the latest European Health Interview Survey (EHIS), 62% of males and 46% of females were overweight, whereas 18% of males and almost 16% of females were obese [3]. Based on the data from the DEGS1 study conducted in Germany, approximately 5.2% of women and 3.9% of men have obesity grade 2, while 2.8% of women and 1.2% of men have obesity grade 3 [4]. A total of 67% of men and 53% of women are overweight, and 23% of men and 24% of women are obese [4].

Obesity is associated with a myriad of serious health consequences and comorbidities, such as type 2 diabetes [5], stroke [6], cardiovascular diseases [7], certain neoplasms [8], and all-cause mortality [9]. Moreover, it has also been proven that patients with excess body mass generate increased healthcare costs [10,11].

There is no doubt that the cornerstones of obesity treatment are diet, physical activity, and behavioral changes. However, lifestyle interventions are often limited in their effectiveness [12], and the majority of obese people struggle to maintain clinically meaningful weight reduction [13]. There is a growing consensus on the role of patient adherence as being the health-related behavior that may influence, either directly or indirectly, satisfactory long-term outcomes of such treatment [14].

Acting in accordance with medical recommendations, including systematically taking medications, is referred to as “compliance”. Adherence, on the other hand, is defined by World Health Organization (WHO) more widely as “a set of health-related behaviors that are not limited to the degree of compliance to prescribed medications, including the agreement with recommendations from a healthcare provider such as following a diet and achieving lifestyle changes” [15].

There are many factors responsible for poor adherence, and it is concluded that identifying all barriers to following medical recommendations, individual patient attitudes, and knowing each therapeutic step are the most important elements influencing adherence [16]. Obese patients may differ in having good/poor social support, namely a barrier, their acceptance/denial of weight gain, namely an attitude, and their consciousness about health status, namely knowledge. Even the fact of being morbidly obese alone is found to be a kind of barrier towards good adherence. While observing patients during lifestyle modification programs in primary healthcare, Arrebola et al. found that obesity grade 3 was one of the important variables identified as predictive of low adherence [17].

Moreover, patients trying to find an effective obesity treatment may have several characteristics that potentially worsen adherence, such as mental disorders, and comorbidities such as diabetes and arterial hypertension, which profoundly impair adherence, hindering weight loss, and thus reducing patient motivation to lose weight [18,19,20].

It was also shown that in the case of morbidly obese individuals after bariatric interventions, poor adherence to follow-up programs, nutrition recommendations, and lifestyle modifications were responsible for inadequate weight loss, weight regain, and other medical complications [21,22,23].

Various outcomes of nonpharmacological treatment efficacy have been reported in the population with morbid obesity. Unick et al. observed that participants with a BMI over 40 kg/m^2^ recruited to the group with intensive lifestyle intervention had similar adherence (they attended 80% of the treatment sessions over year 1), weight reduction, and a decrease in cardiovascular risk compared with less obese patients [24]. They concluded that a behavioral weight loss program is still an effective option for severely obese individuals [24].

However, even worse outcomes of nonpharmacological treatment have been published. Tonatto-Filho et al. showed that the weight-reducing strategy based on the improvement of eating habits and practice of physical activities fails in 95% of such patients [25].

It should be underlined that the patient’s ability to modify lifestyle, including diet and physical activity, is considered to be a key factor in achieving satisfying weight reduction after both surgical and conservative treatment [26,27]. There is a strong need for a thorough estimation of the psychosocial characteristics of such patients at every stage of morbid obesity treatment.

Especially after bariatric surgery, a good patient–therapist relationship seems to provide benefits not only for the prevention of long-term complications but also for sustained weight reduction. Jennings et al. showed greater weight loss in subjects who had good outpatient adherence after laparoscopic gastric bypass surgery [21].

Good adherence seems to be one of the key factors responsible for satisfactory results of complex obesity treatment. Adherence should be routinely assessed to promptly address potential problems in the treatment of obesity, especially morbid obesity, which is usually associated with more severe deterioration of health. Therefore, the focus of the study was to examine and compare adherence between patients treated for morbid obesity living in Poland and Germany. The additional aim was to investigate the impact of adherence on the efficacy of obesity treatment. Such issues as demographic and clinical factors affecting patient adherence were analyzed.

## 2. Materials and Methods

### 2.1. Study Population

A cross-sectional international multicenter survey design was adopted. In the study group, there were 564 adults diagnosed and treated for morbid obesity between January 2018 and December 2019 at selected healthcare facilities in Germany (210 patients) and Poland (354 patients). All the participants met the following inclusion criteria: history of morbid obesity, current body mass index (BMI) ≥ 30 kg/m^2^, with obesity-related comorbidities, surgically or conservatively treated morbid obesity in the study time horizon, age above 18 years old, and the ability to understand and comply with the study procedures, having resided in Poland or Germany during the study time horizon. Subjects who did not meet the inclusion criteria were excluded. The decision to enroll a subject was made by the attending physician based on the adopted inclusion and exclusion criteria. Before the enrollment, patients were informed about the study objective and conditions and gave their written informed consent to participate in it. The results presented in this article are a part of a project that has been already published [22,23].

### 2.2. Study Technique

For the study, a validated, custom-made questionnaire was applied. It included twenty-nine questions related to adherence (Supplementary Material File S1) [24,25,26].The respondents were asked, e.g., if they followed a slimming diet, experienced the yo-yo effect, used dietary supplements supporting weight loss, smoked cigarettes, controlled the number of calories consumed per day, used a healthy diet, controlled blood glucose concentration, followed the medical recommendations, practiced sports, took any pharmacotherapy related to the treatment of obesity, measured blood pressure themselves at home, or knew any pro-health program related to the prevention of obesity. There were also eleven questions related to basic issues, including, e.g., age, sex, current BMI, vocational education, financial status, and comorbid diseases. The study questionnaire was constructed on the base of professional references and then evaluated by national medical consultants in the field of obesity. It was also pretested on a representative sample of 150 subjects treated for morbid obesity (75 Germans and 75 Poles) to estimate the psychometric properties of the German and Polish versions of the questionnaire. Subsequently, the questions could be revised if needed. As the pretest did not yield any major modifications to the questionnaire, the final study included findings from the pretest.

The estimation of depression, anxiety, and stress was performed with the use of a standardized Depression Anxiety Stress Scale questionnaire in Polish and German, versions (DASS–21) [27]. This questionnaire is a good tool for the assessment of the levels of self-perceived depression, anxiety, and stress in participants aged 14 years and above. The DASS-21 with 21 items was constructed as a shorter version of the DASS-42 questionnaire and includes three parts (subscales): depression (7 items), anxiety (7 items), and stress (7 items). The respondents are asked to report experiences during the previous 7 days. Each part comprises 7 questions and is finally scored from 0 to 21, as responses are scored with a Likert scale ranging from zero (0 = did not apply to me) to three (3 = applied to me very much or most of the time). The following cut-off points are used: no symptoms, mild, moderate, severe, and extremely severe. Polish and German versions of DASS-21 were translated with high validity and internal reliability [28,29].

All patients answered research questions independently. The final study comprised complete questionnaires (100% filled in by the patients). The authors made efforts to provide a comprehensive assessment of adherence. The attending physician (as the leader of the obesity treatment staff) was responsible for the final evaluation of adherence, based on the patient’s responses to the questionnaire and self-estimated relationship with the patient during obesity treatment. The method of handling the information obtained during the survey guaranteed absolute confidentiality as each patient was identified as N.N., so the research project did not violate the Personal Data Protection Act. The authors received approval from the ethics committees of the Charité—Universitätsmedizin Berlin and the Poznan University of Medical Sciences (No. KB 326/19; Poznan University of Medical Sciences Bioethics Committee; issued on 7 March 2019).

Among all the participants a physical examination was carried out, and updated patient medical records were analyzed. Weight, height, and BMI were assessed. The weight (in kilograms) was analyzed using electric scales (TANITA TBF-240, Arlington Heights, AZ, USA). Height measurement was performed with the use of a standard measuring rod, barefoot and in the Frankfort position. To evaluate the obesity treatment, two parameters were calculated: %TWL (percentage of total weight loss) and %EWL (percentage of excess weight loss). The following formulas were used to calculate BMI, %TWL, and %EWL [30]:

(a) BMI: weight (kg)/height (m^2^);
(b) %TWL: [(initial weight − current weight)/(initial weight)] × 100;
(c) %EWL: [(initial weight − current weight)/(initial weight –ideal weight)] × 100.

%EWL variables were created, with the ideal weight taken from the tables for Poles [31] and Germans [32].

### 2.3. Statistical Analysis

Means and standard deviations were used to present quantitative parameters, whereas counts and percentages were used to describe categorical data. The Student’s *t*-test was used, or, when data did not follow the normal distribution (Shapiro–Wilks test), the Mann–Whitney test was used to compare interval data between both groups. To analyze categorical data, the Chi-square test for independence was used. A logistic regression analysis was performed to find potential factors influencing patient adherence and study parameters. Furthermore, multiple logistic regression with stepwise, backward selection was used to find an optimal model. The results are presented as odds ratios (ORs) and 95% confidence intervals (95%CIs). The analysis was carried out with the use of the TIBCO Software Inc statistical package (Palo Alto, CA, USA) (2017) Statistica (data analysis software system), version 13. http://statistica.io (accessed on 15 May 2022). All tests were considered significant at *p* < 0.05.

## 3. Results

### 3.1. Characteristics of the Study Group

The sociodemographic characteristics of the study group are shown in Table 1. On the basis of the adopted inclusion criteria, a sample of 564 adults (210 German patients and 354 Polish patients) who were treated for morbid obesity was selected. There were no statistically significant differences between the participants from either country in terms of age, sex, baseline BMI, degree of obesity, duration of the disease, material status, and level of education (*p* > 0.05). Compared to Polish patients, the group of German patients who underwent bariatric procedures was statistically more numerous (*p* < 0.001). A total of 27.48% of study participants (73 Germans and 82 Poles) underwent bariatric surgery, whereas 72.52% of them (137 Germans and 272 Poles) were treated conservatively. In both groups of patients, the most common comorbidities were: arterial hypertension (47.61% and 45.48% of German and Polish participants, respectively), type 2 diabetes (28.57% and 12.71%), coronary heart disease (24.76% and 24.85%), and lipid disorders (19.04% and 17.79%).

### 3.2. Comparison of Adherence between Patients Treated for Morbid Obesity Living in Poland and Germany

The degree of adherence was higher, but statistically insignificant, among Polish patients (83.82% vs. 78.33%, *p* = 0.100). Most participants from both countries admitted that they had been on a reducing diet at least once in their lifetime (86.03% vs. 91.67%, *p* = 0.045) and had experienced the yo-yo effect (repeated cycle of weight loss followed by weight gain, 91.45% vs. 95.45%, *p* = 0.073). Detailed information on adherence was provided in Table 2.

The surveyed Poles more frequently than the Germans tried to use a slimming diet (13.24 times vs. 9.1 times).

Over 18% of Polish patients declared they used supplements for weight reduction. The most popular dietary supplements were the following preparations: white mulberry (36%), a composition of guarana, synephrine, and the Sinetrol complex (32%), and young barley (24%). Over 8% of German participants also used supplements for weight reduction. They most often mentioned supplements, such as a composition of β-1,4-polymer of D-glucosamine and N-acetyl-D-glucosamine, vegetable cellulose, vitamin C, tartaric acid, silicon dioxide, magnesium stearate (40%), a composition of KioSlim ™ complex with KiOnutrime-Csg ^®^, magnesium stearate and hypromellose (30%), and a composition of soy protein, milk protein, vitamins B1, B12, pantothenic acid, iodine, iron, and amino acids (10%). 

Almost two-thirds of Polish patients and almost half of German ones were prescribed medications for any reason. The most popular drugs taken by Poles were: L-thyroxine (33.72%) and metformin (27.91%), whereas those most often taken by Germans were: L-thyroxine (30.36%), ramipril (19.64%), and metformin (14.29%).

Three times more Polish patients, compared to German respondents, admitted to regularly measuring their blood pressure at home.

Polish participants measured glucose concentration over five times more often, compared to German ones (Table 2).

Germans statistically significantly more often practiced sports (*p* < 0.001) (Table 2). They most often had physical activity twice a week (34.15%) or three times a week (30.49%). More than half of them attended the gym (52.44%), while among Poles this percentage was almost two times lower (28.17%). Polish respondents most often practiced sports three times a week (30.99%) or twice a week (16.90%).

Both in Poland and Germany, the physician was a key person responsible for the therapy of obesity (Table 2). The dietitian dealt with the treatment of 37.50% obese patients, both from Poland and Germany. The psychologist treated obesity more than twice as often among German respondents (13.33%) than among Polish ones (5.15%). In Germany, the physiotherapist participated in obesity treatment three times more often (5.00%) than in Poland (1.47%).

### 3.3. Influence of Adherence to Medical Recommendations on Weight Loss and Mental Health of Obese People

The conducted study showed statistically significant differences in the level of reduction in TWL and EWL depending on adherence to medical recommendations in both Polish and German groups. Both the percentage of TWL and EWL were significantly higher in the group of adherent patients compared to the nonadherent patients (% TWL: 16.71 ± 9.64 vs. 4.91 ± 5.46—Poland; 21.05 ± 12.69 vs. 4.83 ± 7.63—Germany; *p* < 0.00001) (% EWL: 23.89 ± 18.15 vs. 7.21 ± 7.19—Poland; 30.27 ± 21.76 vs. 6.30 ± 12.49—Germany; *p* < 0.0001). Moreover, statistically significant differences were observed in the level of perceived anxiety, stress, and depression depending on adherence to medical recommendations in both Polish and German groups of respondents. The analyzed parameters were significantly higher in the group of nonadherent patients (level of depression: 10.79 ± 6.97 vs. 25.24 ± 9.80—Poland; 8.17 ± 6.94 vs. 22.81 ± 11.34—Germany; *p* < 0.00001) (level of anxiety: 7.35 ± 6.21 vs. 16.63 ± 9.69—Poland; 6.18 ± 5.60 vs. 15.24 ± 10.05—Germany; *p* < 0.00001) (level of stress: 12.01 ± 7.59 vs. 22.31 ± 9.81—Poland; 11.35 ± 8.88 vs. 23.33 ± 10.99—Germany; *p* < 0.00001) (Table 3).

### 3.4. Logistic Regression Analysis for Confounders Influencing Patient Adherence

The performed logistic regression analysis found that both in the group of obese participants from Poland and Germany, patient adherence was associated with gender, age, level of education, duration of obesity, number of health professionals involved in obesity treatment, and the type of applied therapy (*p* < 0.05) (Table 4). However, the level of patient adherence showed no significant dependence on the BMI classification. Being an obese man contributed to a significantly higher level of adherence. A positive correlation was observed in the case of age, level of education, and a growing number of health professionals involved in obesity treatment, whereas a negative correlation was observed in the case of the duration of obesity, level of depression, anxiety, and stress. In both groups of respondents, it was observed that the deteriorating mental health of the patient contributes to nonadherence. Moreover, in both countries, patients who underwent bariatric surgery significantly more often followed medical recommendations regarding lifestyle changes compared to obese patients treated only conservatively (Table 4).

Based on the conducted studies, it was observed that in both groups, adherence significantly influenced the effectiveness of the treatment (Table 4). The logistic regression model confirmed that adherence significantly increased %TWL and %EWL due to applied obesity treatment among patients from both countries (*p*< 0.001) (Table 4).

### 3.5. Multiple Logistic Regression Analysis for Confounders Influencing Patient Adherence

The performed multiple logistic regression analysis for confounders influencing patient adherence found that in the Polish group, patient adherence was associated with % TWL (OR = 1.17; CI = 1.09, 1.26), the severe depression level (OR = 0.09; CI = 0.03, 0.33), extremely severe depression level (OR = 0.02; CI = 0.003, 0.133), severe stress level (OR = 0.17; CI = 0.03, 0.86), and obesity grade 3 (OR = 0.36; CI = 0.16, 0.81) (Table 5).

In turn, in the group from Germany, patient adherence was associated with %TWL (OR = 1.42; CI = 1.18, 1.72), %EWL (OR = 1.19; CI = 1.05, 1.33), the extremely severe depression level (OR = 0.08; CI = 0.01, 0.68), and type of obesity treatment (OR = 6.35; CI = 1.68, 23.95) (Table 6).

## 4. Discussion

The presented study is innovative because it is one of the first international reports to provide detailed information on adherence to therapeutic recommendations by patients treated for morbid obesity in Germany and Poland. Comparing the results from two European countries adds value to this study and allows other countries to be advised on how to improve the long-term outcomes of morbid obesity treatment. 

There is no doubt that good adherence to medical recommendations is necessary for the long-lasting success of weight reduction. Many studies identified possible indicators of worse adherence to recommendations, such as demographic variables, medical comorbidities, and cognitive function [33,34]. In particular, these studies were focused on attendance at follow-up visits, dietary suggestions, and vitamin supplementation [35,36].

In the presented study, patient adherence reached 83.82% in the Polish group and 78.33% in the German group (*p* = 0.100). Similar percentages were also reported in studies on therapeutic adherence in obese patients [37,38], while some researchers found that even the vast majority of obese people did not follow medical recommendations, highlighting patients with 50–95% nonadherence [39,40].

### 4.1. Confounders Influencing Patient Adherence

#### 4.1.1. Age and Gender

The evidence reviewed suggests that sociodemographic characteristics such as age and gender, among others, are related to adherence and final results of antiobesity treatment [41,42,43]. This coincides with the presented findings, as a positive correlation was observed in the case of age and gender. Being an obese male subject contributed to a significantly higher level of adherence. Lima et al. and Scozzari et al. reported that there was a statistically significant greater loss in % EWL in females and patients older than 50 years old [41,42]. This fact was explained by typical changes for the elderly: a lower metabolic rate and decreased oxidation of fat, as well as weakened lipolytic activity in postmenopausal women who comprised the majority of the group. In addition, lower levels of physical activity are performed in this age group [41].

In turn, Coleman showed that in the long-term follow-up, females had a higher % EWL than males [43]. As far as the association between age and adherence is concerned, Vidal et al. noted that in the population of 263 patients after bariatric procedures, the nonadherence was greater in patients younger than 45 years old and in those with poor weight reduction (<50% EWL) [37]. McVay et al. also confirmed that older age was one of the patient predictors of good follow-up care attendance (so-called “medical attendance”) among individuals after Roux-en-Y gastric bypass (RYGB). %EWL at 6 months predicted medical attendance at 1 year after bariatric surgery [44].

#### 4.1.2. The Duration of the Disease

In the presented study, a negative correlation was observed in the case of duration of obesity, which is in line with the observations noted by Firth et al., who reported that long-term physical and mental diseases worsen patient adherence [45].

#### 4.1.3. The Level of Education, Employment Status, Material Status, and BMI Classification

Like in the presented study, some researchers showed an association between obesity treatment and such parameters as a level of education [46], material status, and social class [47]. However, Kuzmar et al. did not confirm that these parameters were factors influencing the results of antiobesity treatment [40]. Hadžiabdić et al. recruited 124 obese individuals to a 12-month weight reduction program [48]. They reported that one-third of participants were successful because they reduced their initial weight by more than 5% after the 12-month intervention. Initial weight loss and marital status were the strongest predictors of weight loss success after 1 year. Participants more likely to drop out were those with a lower educational level and a higher obesity grade [48]. In the presented study, the level of patient adherence showed no significant dependence on obesity grades (BMI classification).

Larjani et al. aimed to find patient factors associated with adherence to follow-up care after bariatric procedures (gastric bypass in 91.8% of subjects, sleeve gastrectomy in 8.2%) [49]. They defined adherence as having attended three or four out of four clinic visits. In a population of 388 Canadian participants, they showed that employment was the strongest predictor of attendance at follow-up clinics. Individuals with full-time or part-time employment had a significantly higher adherence rate than those who were unemployed or retired [49].

#### 4.1.4. The Number of Health Professionals Involved in Obesity Management

There is a strong need for a multidisciplinary team for coordinated obesity treatment. In the presented study, the number of health professionals involved in obesity management was related to adherence. In the literature, it is also indicated that an important step towards successful weight reduction is creating a net of healthcare professionals involved in obesity management, including psychologists, social workers, and educators [50,51]. The obesity management team should be well-educated on many aspects of obesity and related comorbidities and psychosocial problems. Solsky et al. aimed to estimate whether an educational intervention could improve physician recognition of patient needs and increase adherence to established best practices for all morbidly obese operatively treated patients [52]. A care map outlining these recommendations was distributed to surgeons and anesthesiologists. This intervention had a significant impact on the percentage of physicians who reported changing their management to match best practices [52].

It was also stressed that if the obesity team increases patients’ knowledge of health problems, the final results of antiobesity treatment are better [53,54]. A Chinese study of overweight or obese people showed that an increase in nutritional knowledge predicted better dietary adherence. At the same time, greater improvements in self-efficacy in physical activity predicted higher adherence to physical activity recommendations [54].

#### 4.1.5. The Level of Depression, Anxiety, and Stress

In the presented study, a negative correlation was shown in the case of levels of depression, anxiety, and stress. This is in line with the findings from a Spanish study that recruited 761 obese patients [55]. These authors noted that initial and final weight and BMI were higher among participants with more severe anxiety or depression. In the population with milder psychiatric disorders, the percentage of weight loss and adherence to therapy was greater [55].

Both in the Polish and German groups of participants, the deteriorating mental health of patients contributed to nonadherence. This is consistent with the observation made by McVay et al., who reported that low anxiety levels predicted good follow-up medical attendance in the population of patients after RYGB [44]. Marek et al. also examined a population of 498 RYGB patients, mostly females, Caucasians, and middle-aged, with the use of the Minnesota Multiphasic Personality Inventory-2-Restructured Form (MMPI-2-RF) [56]. They found that scales from the Behavioral/Externalizing Dysfunction (BXD) domain of the MMPI-2-RF were associated with worse weight loss outcomes and poor adherence to follow-up [56].

It is worth emphasizing that people after bariatric procedures reported depressive symptoms more frequently than subjects treated conservatively [57,58], although lower frequency [59] and no group differences [60] were also reported.

Regardless of how excess body weight is treated, obesity itself greatly increases the odds of any mood and anxiety disorders [61,62,63]. Interesting findings come from the HUNT-2 study of 65 thousand obese adults [61]. There was a positive relationship between abdominal fat distribution and the prevalence of both anxiety and depression, and a negative correlation between BMI and anxiety both in females and males [61]. Petry et al. analyzed the data from 41,654 respondents in the National Epidemiologic Survey on Alcohol and Related Conditions [62]. They found out that after controlling for demographics, the continuous variable of BMI was significantly associated with most mood, anxiety, and personality disorders [62]. In turn, Zhao et al. showed that the age-adjusted prevalence of current depression, lifetime diagnosed depression, and anxiety varied significantly by gender [63]. Psychiatric problems were significantly more often detected in women who were overweight or obese and in morbidly obese males than in people with a normal BMI. After adjusting for demographics, comorbidities, lifestyle, or psychosocial factors, compared with men with a normal BMI, severely obese men were significantly more likely to have current depression or lifetime diagnosed depression and anxiety. Women who were either overweight or obese were significantly more likely than women with a normal BMI to have all three psychiatric disorders [63].

#### 4.1.6. The Type of Antiobesity Therapy Used (Bariatric or Conservative)

In the presented study, participants who underwent bariatric procedures significantly more often followed medical recommendations regarding lifestyle changes compared to obese patients treated only conservatively. This could be partially explained by the individual approach of patients to the treatment. Several factors, including motivation for lifestyle modifications, weight loss goals, and individual beliefs about achieving success in the final weight reduction, are found to be associated with adherence to obesity treatment recommendations, especially after bariatric procedures [64]. Kvalem et al. aimed to compare the behavioral and psychological characteristics of severely obese individuals opting for bariatric or conservative treatment [65]. Authors found that candidates for bariatric surgery had more positive expectations of outcomes and believed more strongly they would succeed in losing weight, whereas patients starting conservative treatment were more convinced of their readiness to improve their physical activity [65].

### 4.2. The Impact of Adherence on the Efficacy of Obesity Treatment

Based on the conducted studies, it was observed that adherence significantly increased %TWL and %EWL, and that %TWL and %EWL were significantly higher in the group of adherent patients compared to the nonadherent patients. These results are in line with the studies by Sarwer et al. and Wakayama et al. [53,66]. Sarwer et al. reported that low dietary adherence at 6 months after bariatric procedures predicted lower weight loss [53]. Wakayama et al. confirmed that 6-month postoperative dietary adherence predicts 12-month BMI, %EWL, and %TWL [66]. 

A % EWL greater than 50% is considered adequate [67]. Luca et al. conducted a retrospective, single-center, cohort study, including all patients undergoing bariatric procedures [38]. At 3 and 5 years after surgery, among adherent patients, %EWL was 73.6% and 81.2%, respectively, while among nonadherent subjects, it was 70.7% and 68.4%, respectively. However, the difference between adherent and nonadherent groups was nonsignificant [38]. Vidal et al. aimed to estimate adherence after bariatric procedures and analyzed data of 263 such patients [37]. Nonadherence was 17.5% and was defined as missing any scheduled control visit for more than half a year [37].

The levels of perceived anxiety, stress, and depression were significantly higher in nonadherent patients in both countries. In both examined groups, patient adherence was associated with % TWL and the extremely severe depression level in the Polish population, as well as with the severe depression level, severe stress level, and obesity grade 3, whereas in the German population, it was additionally associated with the type of obesity treatment. One of the possible explanations for the discrepancies is that Polish and German patients are treated in different healthcare systems. In Germany, there are several programs devoted to the prevention and treatment of obesity, while no such programs are currently available for Polish patients, who are often alone in the stressful fight against excess weight. German patients also have better access to bariatric procedures. Worldwide, there is a need for personalized strategies in designing follow-up programs for patients treated for obesity, especially grade 3. In a Chinese study that was conducted on 288 adults after bariatric procedures, Zhu et al. determined that the factors with the greatest effects on adherence included attitude, intention, time since surgery, exercise, social influence, and self-efficacy [68]. A meta-analysis performed by Burgess et al. showed that behavioral treatment interventions, including, e.g., goal setting, motivational interviewing, and cognitive restructuring, have a significant positive effect on adherence to treatment in adults with obesity [69].

In a study dedicated to the reasons for nonadherence among patients with morbid obesity after bariatric surgery, Luca et al. revealed that almost one-third of people were lost to follow-up [38]. After a callback, data were collected from 89.9% of patients. A total of 62.9% of reasons for nonattendance were related to personal matters, occupational matters, health problems, and dissatisfaction with poor weight loss [38]. This also stresses the need for a multidisciplinary obesity team, including psychologists, psychiatrists, and social workers.

Contributing to this line of research, for the first time among Polish and German patients, we examined the importance of good adherence to the recommendations in obesity treatment. Our logistic regression model showed that adherence significantly increased %TWL and %EWL due to applied obesity treatment among patients, both from Germany and Poland.

### 4.3. Study Limitations

The presented study is limited in several aspects. The authors are aware that although German and Polish participants completed the same questionnaires, some issues may be differently understood due to specific environmental and social factors, including differences in the support of public health for morbidly obese individuals. Moreover, the study population consisted mainly of middle-aged and elderly patients. Further research is needed to explore if similar findings might be observed in the younger population. The presented research sample was identified as white, so it should be verified whether our results can be extended to a different ethnic group. 

There are discrepancies between clinician and patient perceptions of adherence. For example, calorie intake was proved to be underreported among patients seeking to lose weight, especially among those with a higher BMI [70], and self-monitoring adherence was also frequently overreported [71,72,73,74]. 

What is more, there could be also problems in the therapeutic relationship—for instance, a patient’s reluctance to be honest with a physician or a physician’s failure to establish a strong rapport with a patient—which may result in bad long-term weight outcomes [75,76].

Berry MP et al. showed that ratings of adherence were higher when reported by patients and supported the hypothesis that patients who provided higher adherence ratings relative to their physicians lost less weight during treatment (*p* < 0.001) [77]. The authors concluded that those participants who frequently appraise their adherence more highly than their clinicians are at a greater risk of lower weight reduction [77].

Therefore, the authors wanted to take all these aspects into account and made efforts to objectify the assessment as much as possible.

## 5. Conclusions

In the presented study, no statistically significant difference was found in the general level of adherence in the population of patients treated for morbid obesity from Poland and Germany. In both countries, adherence was found to be related to gender, age, education level, duration of obesity, and the type of therapy used (bariatric or conservative). Patients after bariatric surgery significantly more often complied with medical recommendations regarding lifestyle changes compared to obese patients treated only conservatively.

Our most significant and innovative finding was that the number of health professionals involved in obesity management was related to adherence. This indicates the need to build a network of professional teams that will be dedicated to the long-term follow-up of patients treated for morbid obesity.

Both in Polish and German groups, patient adherence increased %TWL and %EWL. % TWL and % EWL were significantly higher in the group of adherent patients compared to the nonadherent patients. The levels of perceived anxiety, stress, and depression were significantly higher in nonadherent patients in both countries.

Taken together, the presented findings underline the role of good adherence in the effective treatment of morbid obesity. As a result, there is a great need to increase patient adherence and hence improve obesity treatment outcomes worldwide.

## Figures and Tables

**Table 1:** Sociodemographic and clinical parameters of obese patients from Poland and Germany (*n* = 564).

Variables	Variables	Poland	Germany
Group size	Total	354	210
	Female %	77.40	75.24 *p* = 0.7563 ^a^
	Male %	22.60	24.76 *p* = 0.5369 ^a^
	Surgically treated patients *n* (%)	82 (23.16)	73 (34.76)
	Conservatively treated patients *n* (%)	272 (76.84)	137 (65.24)
Age (years)	Mean ± SD	45.20 ± 15.69	45.70 ± 9.70 *p* = 0.8541 ^a^
Body mass index—BMI [kg/m^2^]	Mean ± SD	36.92 ± 8.12	36.87 ± 10.06 *p* = 0.9452 ^a^
Obesity grade 1 (30–34.9 kg/m^2^)	%	27.96	27.14 *p* = 0.8876 ^a^
Obesity grade 2 (35–39.9 kg/m^2^)	%	29.66	25.24 *p* = 0.0681 ^a^
Obesity grade 3 (≥40 kg/m^2^)	%	42.38	47.76 *p* = 0.0741 ^a^
Duration of the disease (years)	Mean ± SD	17.67 ± 11.61	17.00 ± 10.51 *p* = 0.7452 ^a^
Vocational education	Low level (%)	36.44	36.71 *p* = 0.8941 ^a^
	Average level (%)	42.66	41.06 *p* = 0.7452 ^a^
	High level (%)	20.90	22.23 *p* = 0.1237 ^a^
Material status	Definitely good (%)	10.20	10.50 *p* = 0.9456 ^a^
	Good (%)	34.36	35.66 *p* = 0.5632 ^a^
	Average (%)	42.66	41.06 *p* = 0.6569 ^a^
	Bad (%)	7.63	7.11 *p* = 0.8962 ^a^
	Definitely bad (%)	4.19	2.57 *p* = 0.0956 ^a^
Comorbidities	Type 2 diabetes mellitus (%)	27.11	28.57 *p* = 0.4589 ^a^
	Arterial hypertension (%)	45.48	47.61 *p* = 0.1253 ^a^
	Dyslipidemia (%)	17.79	19.04 *p* = 0.1478 ^a^
	Hyperuricemia (%)	3.95	5.71 *p* = 0.0635 ^a^
	Metabolic syndrome (%)	13.56	16.66 *p* = 0.0856 ^a^
	Coronary heart disease (%)	24.85	24.76 *p* = 0.7563 ^a^
Type of bariatric surgery	Gastric balloon (%)	1.22	0
	Laparoscopic adjustable gastric banding (%)	6.10	0
	Laparoscopic Roux-en-Y gastric bypass (%)	10.98	54.79 *p* < 0.001
	Laparoscopic sleeve gastrectomy (%)	81.70	45.21 *p* < 0.001

^a^. statistically significant difference: Poland versus Germany for *p* < 0.05. Material status categories: Definitely good EUR > 3000 per month; Good EUR 2001–3000 per month; Average EUR 1001–2000 per month; Bad EUR 500–1000 per month; Definitely bad EUR < 500 per month.

**Table 2:** Comparison of adherence between patients treated for morbid obesity living in Poland (*n* = 354) and Germany (*n* = 210).

Variables	Poland (*n* = 354)	Germany (*n* = 210)	*p* Value
Adherent patients (%)	83.82	78.3	*p* = 0.100
Patients who admitted to being on a reduced diet during their lifetime (%)	86.03	91.67	*p* = 0.045
Patients who had experienced the yo-yo effect further during their lifetime (%)	91.45	95.45	*p* = 0.073
Patients who used supplements during weight reduction (%)	18.38	8.33	*p* = 0.001
Patients who admitted to being current smokers (%)	21.32	19.17	*p* = 0.541
Current smokers who declared the will to quit cigarette smoking (%)	51.72	60.87	*p* = 0.034
Patients who count calories during everyday life (%)	31.62	23.33	*p* = 0.035
Patients who declared that they eat healthy (%)	41.91	49.17	*p* = 0.093
Patients who declared regular taking pharmacotherapy for any reason (%)	63.24	46.67	*p* <0.001
Patients who declared regular measuring blood pressure at home (%)	62.50	20.00	*p* < 0.001
Patients who declared measuring blood pressure at home once a day (%)	34.12	4.17	*p* < 0.001
Patients who declared regular measuring glucose levels at home (%)	33.09	5.83	*p* < 0.001
Patients who declared regular physical activity (%)	52.21	68.33	*p* < 0.001
Patients who declared regular physical activity three times a week (%)	30.99	30.49	*p* = 0.901
Active physically patients who pay for thy gym on their own (%)	100.00	83.72	*p* < 0.001
Patients who declared that if they had the opportunity to attend sports activities for free, would participate in them (%)	78.68	89.17	*p* = 0.001
Patients who declared that a physician is a key person responsible for their obesity treatment (%)	74.26	87.50	*p* < 0.001

**Table 3:** Influence of adherence to medical recommendations on weight loss and mental health of morbidly obese patients in Poland (*n* = 354) and Germany (*n* = 210).

Variables	Poland			Germany		
	Adherent Patients (mean ± SD)	Nonadherent Patients (mean ± SD)	*p* Value	Adherent Patients (mean ± SD)	Nonadherent Patients (mean ± SD)	*p* Value
% TWL	16.71 ± 9.64	4.91 ± 5.46	<0.00001	21.05 ± 12.69	4.83 ± 7.63	<0.00001
% EWL	23.89 ± 18.15	7.21 ± 7.19	<0.00001	30.27 ± 21.76	6.30 ± 12.49	<0.00001
Level of depression	10.79 ± 6.97	25.24 ± 9.80	<0.00001	8.17 ± 6.94	22.81 ± 11.34	<0.00001
Level of anxiety	7.35 ± 6.21	16.63 ± 9.69	<0.00001	6.18 ± 5.60	15.24 ± 10.05	<0.00001
Level of stress	12.01 ± 7.59	22.31 ± 9.81	<0.00001	11.35 ± 8.88	23.33 ± 10.99	<0.00001

**Table 4:** Logistic regression analysis for confounders influencing patient adherence to obesity treatment in Poland (*n* = 354) and Germany (*n* = 210).

		Poland *n* = 354	Germany *n* = 210			
Variable	OR	95% CI	*p* Value	OR	95% CI	*p* Value
Sex:						
Female	1.0 (ref *)			1.0 (ref)		
Male	1.39	0.83, 2.33	0.199	2.34	1.19, 4.61	0.014
Age	1.03	1.01, 1.05	0.001	2.08	1.15, 3.74	0.015
Education:						
Primary	1.0 (ref)			1.0 (ref)		
Vocational	1.13	0.34, 3.74	0.841	1.25	0.29, 5.36	0.764
Secondary	1.66	0.53, 5.22	0.386	1.42	0.30, 6.68	0.656
Higher	2.18	0.62, 7.61	0.219	2.77	1.37, 5.60	0.004
Duration of obesity	0.95	0.93, 0.98	0.001	0.56	0.40, 0.80	0.001
Number of health professionals involved in obesity treatment	1.95	1.18, 3.26	0.010	1.15	0.80, 1.65	0.449
% TWL (percentage of total weight loss)	1.33	1.24, 1.43	<0.001	1.22	1.15, 1.29	<0.001
% EWL (percentage of excess weight loss)	1.19	1.14, 1.25	<0.001	1.13	1.09, 1.18	<0.001
Type of obesity treatment: Conservative Bariatric surgery						
	1.0 (ref)			1.0 (ref)		
	25.91	9.22, 72.75	<0.001	26.75	9.77, 73.18	<0.001
BMI classification:						
Obesity grade 1	1.0 (ref)			1.0 (ref)		
Obesity grade 2	0.56	0.23, 1.34	0.197	0.61	0.24, 1.54	0.301
Obesity grade 3	0.49	0.21, 1.19	0.116	0.58	0.21, 1.60	0.300
Level of depression:						
Normal	1.0 (ref)			1.0 (ref)		
Mild	0.86	0.29, 2.53	0.86	0.73	0.23, 2.29	0.591
Moderate	0.21	0.10, 0.47	<0.001	0.39	0.16, 0.92	0.033
Severe	0.01	0.01, 0.04	<0.001	0.01	0.004, 0.09	<0.001
Extremely severe	0.007	0.01, 0.02	< 0.001	0.01	0.001, 0.04	<0.001
Level of anxiety:						
Normal	1.0 (ref)			1.0 (ref)		
Mild	0.66	0.29, 1.50	0.327	0.46	0.15, 1.43	0.183
Moderate	0.33	0.18, 0.63	0.001	0.32	0.14, 0.69	0.004
Severe	0.16	0.07, 0.34	<0.001	0.15	0.05, 0.46	<0.001
Extremely severe	0.035	0.01, 0.08	<0.001	0.02	0.01, 0.08	0.001
Level of stress:						
Normal	1.0 (ref)			1.0 (ref)		
Mild	0.26	0.13, 0.52	<0.0001	0.17	0.06, 0.47	<0.0001
Moderate	0.16	0.08, 0.32	<0.0001	0.24	0.11, 0.56	<0.0001
Severe	0.08	0.04, 0.17	<0.0001	0.05	0.02, 0.14	<0.0001
Extremely severe	0.01	0.001, 0.07	<0.0001	0.01	0.001, 0.116	<0.0001

*—reference group

**Table 5:** Multiple logistic regression model results for the risk factor of adherence in the field of obesity treatment in Poland (*n* = 354).

Variable		Poland *n* = 354	
	OR	95% CI	*p* Value
% TWL (percentage of total weight loss)	1.17	1.09, 1.26	<0.0001
Depression level: Severe Extremely severe	0.09 0.02	0.03, 0.33 0.003, 0.133	<0.0001 < 0.0001
Stress level: Severe	0.17	0.03, 0.86	0.032
BMI classification: Obesity grade 3	0.36	0.16, 0.81	0.013

**Table 6:** Multiple logistic regression model results for the risk factor of adherence in the field of obesity treatment in Germany (*n* = 210).

Variable		Germany *n* = 210	
	OR	95% CI	*p* Value
% TWL (percentage of total weight loss)	1.42	1.18, 1.72	<0.0001
% EWL (percentage of excess weight loss)	1.19	1.05, 1.33	0.004
Depression level: Extremely severe	0.08	0.01, 0.68	0.021
Type of obesity treatment: Conservative Bariatric surgery	1.0 (ref) 6.35	1.68, 23.95	0.006

## Data Availability

The datasets used and/or analyzed during the current study are available from the corresponding author on reasonable request.

## Supplementary Materials

The following supporting information can be downloaded at: https://www.mdpi.com/article/10.3390/nu14183880/s1, File S1: The study questionnaire.

## References

[1] World Health Organization (2015). Global Health Observatory Data. www.who.int/ghoNCDd/risk_factors/overweight/en/.

[2] Hales C.M., Carroll M.D., Fryar C.D., Ogden C.L. (2020). Prevalence of Obesity and Severe Obesity among Adults: The United States, 2017–2018. NCHS Data Brief.

[3] Eurostat European Health Interview Survey. https://ec.europa.eu/eurostat/web/microdata/european-health-interview-survey.

[4] Mensink G.B., Schienkiewitz A., Haftenberger M., Lampert T., Ziese T., Scheidt-Nave C. (2013). Übergewicht und Adipositas in Deutschland: Ergebnisse der Studie zur Gesundheit Erwachsener in Deutschland (DEGS1) [Overweight and obesity in Germany: Results of the German Health Interview and Examination Survey for Adults (DEGS1)]. Bundesgesundheitsblatt Gesundh. Gesundh..

[5] Kodama S., Horikawa C., Fujihara K., Yoshizawa S., Yachi Y., Tanaka S., Ohara N., Matsunaga S., Yamada T., Hanyu O. (2014). Quantitative relationship between body weight gain in adulthood and incident type 2 diabetes: A meta-analysis. Obes. Rev..

[6] Suk S.H., Sacco R.L., Boden-Albala B., Cheun J.F., Pittman J.G., Elkind M.S., Paik M.C., Northern Manhattan Stroke Study (2003). Abdominal obesity and risk of ischemic stroke: The Northern Manhattan Stroke Study. Stroke.

[7] Nakamura K., Fuster J.J., Walsh K. (2014). Adipokines: A link between obesity and cardiovascular disease. J. Cardiol..

[8] Moghaddam A.A., Woodward M., Huxley R. (2007). Obesity and risk of colorectal cancer: A meta-analysis of 31 studies with 70,000 events. Cancer Epidemiol. Biomark. Prev..

[9] Flegal K.M., Kit B.K., Orpana H., Graubard B.I. (2013). Association of all-cause mortality with overweight and obesity using standard body mass index categories: A systematic review and meta-analysis. JAMA.

[10] Lehnert T., Sonntag D., Konnopka A., Riedel-Heller S., König H.H. (2013). Economic costs of overweight and obesity. Best Pract. Res. Clin. Endocrinol. Metab..

[11] Dobbs R., Sawers C., Thompson F., Manyika J., Woetzel J., Child P., McKenna S., Spatharou A. (2014). Overcoming Obesity: An Initial Economic Analysis. McKinsey Global Institute.

[12] Burguera B., Jesús Tur J., Escudero A.J., Alos M., Pagán A., Cortés B., González X.F., Soriano J.B. (2015). An Intensive Lifestyle Intervention Is an Effective Treatment of Morbid Obesity: The TRAMOMTANA Study-A Two-Year Randomized Controlled Clinical Trial. Int. J. Endocrinol..

[13] Mann T., Tomiyama A.J., Westling E., Lew A.M., Samuels B., Chatman J. (2007). Medicare’s search for effective obesity treatments: Diets are not the answer. Am. Psychol..

[14] Adler S., Fowler N., Robinson A.H., Salcido L., Darcy A., Toyama H., Safer D.L. (2018). Correlates of Dietary Adherence and Maladaptive Eating Patterns Following Roux-en-Y Bariatric Surgery. Obes. Surg..

[15] World Health Organization (WHO) (2003). Adherence to Long-Term Therapies: Evidence for Action/[Edited by Eduardo Sabaté]. World Health Organization.

[16] Wu J.R., Chung M., Lennie T.A., Hall L.A., Moser D.K. (2008). Testing the psychometric properties of the Medication Adherence Scale in patients with heart failure. Heart Lung J. Crit. Care.

[17] Arrebola Vivas E., López Plaza B., Koester Weber T., Bermejo López L., Palma Milla S., Lisbona Catalán A., Gómez-Candela C. (2013). Variables predictoras de baja adherencia a un programa de modificación de estilos de vida para el tratamiento del exceso de peso en atención primaria [Predictor variables for low adherence to a lifestyle modification program of overweight treatment in primary health care]. Nutr. Hosp..

[18] Thereaux J., Lesuffleur T., Czernichow S., Basdevant A., Msika S., Nocca D., Millat B., Fagot-Campagna A. (2018). Association Between Bariatric Surgery and Rates of Continuation, Discontinuation, or Initiation of Antidiabetes Treatment 6 Years Later. JAMA Surg..

[19] Mauro M., Taylor V., Wharton S., Sharma A.M. (2008). Barriers to obesity treatment. Eur. J. Intern. Med..

[20] Bianciardi E., Fabbricatore M., Di Lorenzo G., Innamorati M., Tomassini L., Gentileschi P., Niolu C., Siracusano A., Imperatori C. (2019). Prevalence of Food Addiction and Binge Eating in an Italian sample of bariatric surgery candidates and overweight/obese patients seeking low-energy-diet therapy. Riv. Di Psichiatr..

[21] Jennings N., Boyle M., Mahawar K., Balupuri S., Small P. (2013). The relationship of distance from the surgical centre on attendance and weight loss after laparoscopic gastric bypass surgery in the United Kingdom. Clin. Obes..

[22] Paczkowska A., Hoffmann K., Raakow J., Pross M., Berghaus R., Michalak M., Bryl W., Marzec K., Kopciuch D., Zaprutko T. (2022). Impact of bariatric surgery on depression, anxiety and stress symptoms among patients with morbid obesity: International multicentre study in Poland and Germany. BJPsych Open.

[23] Hoffmann K., Paczkowska A., Bryl W., Marzec K., Raakow J., Pross M., Berghaus R., Nowakowska E., Kus K., Michalak M. (2022). Comparison of Perceived Weight Discrimination between Polish and German Patients Underwent Bariatric Surgery or Endoscopic Method versus Conservative Treatment for Morbid Obesity: An International Multicenter Study. Nutrients.

[24] Kwon Y.J., Lee H., Yoon Y., Kim H.M., Chu S.H., Lee J.W. (2020). Development and Validation of a Questionnaire to Measure Adherence to the Mediterranean Diet in Korean Adults. Nutrients.

[25] Luzak A., Heier M., Thorand B., Laxy M., Nowak D., Peters A., Schulz H., KORA-Study Group (2017). Physical activity levels, duration pattern and adherence to WHO recommendations in German adults. PLoS ONE.

[26] Bianciardi E., Imperatori C., Innamorati M., Fabbricatore M., Monacelli A.M., Pelle M., Siracusano A., Niolu C., Gentileschi P. (2021). Measuring Knowledge, Attitudes, and Barriers to Medication Adherence in Potential Bariatric Surgery Patients. Obes. Surg..

[27] Antony M.M., Bieling P.J., Cox B.J., Enns M.W., Swinson R.P. (1998). Psychometric properties of the 42-item and 21-item versions of the Depression Anxiety Stress Scales in clinical groups and a community sample. Psychol. Assess..

[28] Nilges P., Essau C. (2015). Die Depressions-Angst-Stress-Skalen: Der DASS–ein Screeningverfahren nicht nur für Schmerzpatienten [Depression, anxiety and stress scales: DASS—A screening procedure not only for pain patients]. Schmerz.

[29] Zawislak D., Zur-Wyrozumska K., Habera M., Skrzypiec K., Pac A., Cebula G. (2020). Evaluation of a Polish Version of the Depression Anxiety Stress Scales (DASS-21). J. Neurosci. Cogn. Stud..

[30] Baltasar A., Pérez N., Serra C., Bou R., Bengochea M., Borrás F. (2011). Weight Loss Reporting: Predicted Body Mass Index after Bariatric Surgery. Obes. Surg..

[31] Różdżyńska-Świątkowska A., Kułaga Z., Grajda A., Gurzkowska B., Góźdź M., Wojtyło M., Świąder A., Litwin M., OLAF and OLA Research Group (2013). Height, weight and body mass index references for growth and nutritional status assessment in children and adolescents 3–18 year of age. Stand. Med. Pediatr..

[32] (2013). Beiträge zur Gesundheitsberichterstattung des Bundes. Referenzperzentile für Anthropometrische Maßzahlen und Blutdruck aus der Studie zur Gesundheit von Kindern und Jugendlichen in Deutschland (KiGGS) [Reference Percentiles for Anthropometric Measurements and Blood Pressure from the Study on the Health of Children and Adolescents in Germany (KiGGS)], Robert Koch-Institut. https://www.rki.de/DE/Content/Gesundheitsmonitoring/Gesundheitsberichterstattung/GBEDownloadsB/KiGGS_Referenzperzentile.pdf?blob=publicationFile.

[33] Galioto R., Gunstad J., Heinberg L.J., Spitznagel M.B. (2013). Adherence and weight loss outcomes in bariatric surgery: Does cognitive function play a role?. Obes. Surg..

[34] Goldenshluger A., Elazary R., Cohen M.J., Goldenshluger M., Ben-Porat T., Nowotni J., Geraisi H., Amun M., Pikarsky A.J., Keinan-Boker L. (2018). Predictors for Adherence to Multidisciplinary Follow-Up Care after Sleeve Gastrectomy. Obes. Surg..

[35] Aarts F., Geenen R., Gerdes V.E., van de Laar A., Brandjes D.P., Hinnen C. (2015). Attachment anxiety predicts poor adherence to dietary recommendations: An indirect effect on weight change 1 year after gastric bypass surgery. Obes. Surg..

[36] Shen S.C., Lin H.Y., Huang C.K., Yen Y.C. (2016). Adherence to Psychiatric Follow-up Predicts 1-Year BMI Loss in Gastric Bypass Surgery Patients. Obes. Surg..

[37] Vidal P., Ramón J.M., Goday A., Parri A., Crous X., Trillo L., Pera M., Grande L. (2014). Lack of adherence to follow-up visits after bariatric surgery: Reasons and outcome. Obes. Surg..

[38] Luca P., Nicolas C., Marina V., Sarah B., Andrea L. (2021). Where Are My Patients? Lost and Found in Bariatric Surgery. Obes. Surg..

[39] Tonatto-Filho A.J., Gallotti F.M., Chedid M.F., Grezzana-Filho T., Garcia A. (2019). Bariatric surgery in brazilian public health system: The good, the bad and the ugly, or a long way to go. Yellow sign!. ABCD. Arq. Bras. De Cir. Dig. (São Paulo).

[40] Kuzmar I., Rizo M., Cortés-Castell E. (2014). Adherence to an overweight and obesity treatment: How to motivate a patient?. PeerJ.

[41] Lima R.C., Rodrigues T.M.D.S., Scheibe C.L., Campelo G.P., Pinto L.E.V., Valadão G.J.C., Carvalho G.P.C., Machado Junior M.R.D., Valadão J.A., Lima P.C.R. (2021). Weight loss and adherence to postoperative follow-up after vertical gastrectomy for obesity treatment. Acta Cir. Bras..

[42] Scozzari G., Passera R., Benvenga R., Toppino M., Morino M. (2012). Age as a long-term prognostic factor in bariatric surgery. Ann. Surg..

[43] Coleman K.J., Huang Y.C., Hendee F., Watson H.L., Casillas R.A., Brookey J. (2014). Three-year weight outcomes from a bariatric surgery registry in a large integrated healthcare system. Surg. Obes. Relat. Dis..

[44] McVay M.A., Friedman K.E., Applegate K.L., Portenier D.D. (2013). Patient predictors of follow-up care attendance in Roux-en-Y gastric bypass patients. Surg. Obes. Relat. Dis..

[45] Firth J., Siddiqi N., Koyanagi A., Siskind D., Rosenbaum S., Galletly C., Allan S., Caneo C., Carney R., Carvalho A.F. (2019). The Lancet Psychiatry Commission: A blueprint for protecting physical health in people with mental illness. Lancet Psychiatry.

[46] Mazure R.A., Cancer E., Martínez Olmos M.A., De Castro M.L., Abilés V., Abilés J., Bretón I., Álvarez V., Peláez N., Culebras J.M. (2014). Adherencia y fidelidad en el paciente tratado con balon intragastrico [Adherence and fidelity in patients treated with intragastric balloon]. Nutr. Hosp..

[47] Da Veiga G.V., da Cunha A.S., Sichieri R. (2004). Trends in overweight among adolescents living in the poorest and richest regions of Brazil. Am. J. Public Health.

[48] Hadžiabdić M.O., Mucalo I., Hrabač P., Matić T., Rahelić D., Božikov V. (2015). Factors predictive of drop-out and weight loss success in weight management of obese patients. J. Hum. Nutr. Diet..

[49] Larjani S., Spivak I., Hao Guo M., Aliarzadeh B., Wang W., Robinson S., Sockalingam S., Aarts M.A. (2016). Preoperative predictors of adherence to multidisciplinary follow-up care postbariatric surgery. Surg. Obes. Relat. Dis..

[50] Sharma A.M., Bélanger A., Carson V., Krah J., Langlois M.F., Lawlor D., Lepage S., Liu A., Macklin D.A., MacKay N. (2019). Perceptions of barriers to effective obesity management in Canada: Results from the ACTION study. Clin. Obes..

[51] Trujillo-Garrido N., Santi-Cano M.J. (2022). Motivation and Limiting Factors for Adherence to Weight Loss Interventions among Patients with Obesity in Primary Care. Nutrients.

[52] Solsky I., Edelstein A., Brodman M., Kaleya R., Rosenblatt M., Santana C., Feldman D.L., Kischak P., Somerville D., Mudiraj S. (2016). Perioperative care map improves compliance with best practices for the morbidly obese. Surgery.

[53] Sarwer D.B., Moore R.H., Spitzer J.C., Wadden T.A., Raper S.E., Williams N.N. (2012). A pilot study investigating the efficacy of postoperative dietary counseling to improve outcomes after bariatric surgery. Surg. Obes. Relat. Dis. Off. J. Am. Soc. Bariatr. Surg..

[54] Leung A., Chan R., Sea M., Woo J. (2020). Psychological Factors of Long-Term Dietary and Physical Activity Adherence among Chinese Adults with Overweight and Obesity in a Community-Based Lifestyle Modification Program: A Mixed-Method Study. Nutrients.

[55] Violante R., Santoro S., González C. (2011). Prevalencia de depresión y ansiedad en una cohorte de 761 obesos: Implicancias en la adherencia al tratamiento y sus resultados [Prevalence of depression and anxiety in a cohort of 761 obese patients: Impact in adherence to therapy and its outcome]. Vertex (Buenos Aires Argentina).

[56] Marek R.J., Tarescavage A.M., Ben-Porath Y.S., Ashton K., Merrell Rish J., Heinberg L.J. (2015). Using presurgical psychological testing to predict 1-year appointment adherence and weight loss in bariatric surgery patients: Predictive validity and methodological considerations. Surg. Obes. Relat. Dis. Off. J. Am. Soc. Bariatr. Surg..

[57] Ahnis A., Figura A., Hofmann T., Stengel A., Elbelt U., Klapp B.F. (2015). Surgically and conservatively treated obese patients differ in psychological factors, regardless of body mass index or obesity-related co-morbidities: A comparison between groups and an analysis of predictors. PLoS ONE.

[58] Castellini G., Godini L., Amedei S.G., Galli V., Alpigiano G., Mugnaini E., Veltri M., Rellini A.H., Rotella C.M., Faravelli C. (2014). Psychopathological similarities and differences between obese patients seeking surgical and non-surgical overweight treatments. Eat. Weight. Disord. EWD.

[59] Rutledge T., Adler S., Friedman R. (2011). A prospective assessment of psychosocial factors among bariatric versus non-bariatric surgery candidates. Obes. Surg..

[60] Gradaschi R., Noli G., Cornicelli M., Camerini G., Scopinaro N., Adami G.F. (2013). Do clinical and behavioural correlates of obese patients seeking bariatric surgery differ from those of individuals involved in conservative weight loss programme?. J. Hum. Nutr. Diet. Off. J. Br. Diet. Assoc..

[61] Rivenes A.C., Harvey S.B., Mykletun A. (2009). The relationship between abdominal fat, obesity, and common mental disorders: Results from the HUNT study. J. Psychosom. Res..

[62] Petry N.M., Barry D., Pietrzak R.H., Wagner J.A. (2008). Overweight and obesity are associated with psychiatric disorders: Results from the National Epidemiologic Survey on Alcohol and Related Conditions. Psychosom. Med..

[63] Zhao G., Ford E.S., Dhingra S., Li C., Strine T.W., Mokdad A.H. (2009). Depression and anxiety among US adults: Associations with body mass index. Int. J. Obes..

[64] Gelinas B.L., Delparte C.A., Hart R., Wright K.D. (2013). Unrealistic weight loss goals and expectations among bariatric surgery candidates: The impact on pre- and postsurgical weight outcomes. Bariatr. Surg. Pract. Patient Care.

[65] Kvalem I.L., Bergh I., von Soest T., Rosenvinge J.H., Johnsen T.A., Martinsen E.W., Mala T., Kristinsson J.A. (2016). A comparison of behavioral and psychological characteristics of patients opting for surgical and conservative treatment for morbid obesity. BMC Obes..

[66] Wakayama L., Nameth K., Adler S., Safer D.L. (2019). Replication and extension of dietary adherence as a predictor of suboptimal weight-loss outcomes in postbariatric patients. Surg. Obes. Relat. Dis. Off. J. Am. Soc. Bariatr. Surg..

[67] Di Lorenzo N., Antoniou S.A., Batterham R.L., Busetto L., Godoroja D., Iossa A., Carrano F.M., Agresta F., Alarçon I., Azran C. (2020). Clinical practice guidelines of the European Association for Endoscopic Surgery (EAES) on bariatric surgery: Update 2020 endorsed by IFSO-EC, EASO, and ESPCOP. Surg. Endosc..

[68] Zhu H., Zhao K., Ren Z., Hua H., Zhang T., Ding L., Jiang X., Yang N., Liang H., Zhu S. (2022). Determinants of Dietary Adherence Among Chinese Patients After Bariatric Surgery Based on the Attitude-Social Influence-Efficacy Model. Obes. Surg..

[69] Burgess E., Hassmén P., Welvaert M., Pumpa K.L. (2017). Behavioural treatment strategies improve adherence to lifestyle intervention programmes in adults with obesity: A systematic review and meta-analysis. Clin. Obes..

[70] Carels R.A., Harper J., Konrad K. (2006). Qualitative perceptions and caloric estimations of healthy and unhealthy foods by behavioral weight loss participants. Appetite.

[71] Macdiarmid J., Blundell J. (1998). Assessing dietary intake: Who, what and why of under-reporting. Nutr. Res. Rev..

[72] Lansky D., Brownell K.D. (1982). Estimates of food quantity and calories: Errors in self-report among obese patients. Am. J. Clin. Nutr..

[73] Burke L.E., Sereika S.M., Music E., Warziski M., Styn M.A., Stone A. (2008). Using instrumented paper diaries to document self-monitoring patterns in weight loss. Contemp. Clin. Trials.

[74] Baker R.C., Kirschenbaum D.S. (1993). Self-monitoring may be necessary for successful weight control. Behav. Ther..

[75] Martin L.R., Williams S.L., Haskard K.B., Dimatteo M.R. (2005). The challenge of patient adherence. Ther. Clin. Risk Manag..

[76] Kelley J.M., Kraft-Todd G., Schapira L., Kossowsky J., Riess H. (2014). The influence of the patient-clinician relationship on healthcare outcomes: A systematic review and meta-analysis of randomized controlled trials. PLoS ONE.

[77] Berry M.P., Seburg E.M., Butryn M.L., Jeffery R.W., Crane M.M., Levy R.L., Forman E.M., Sherwood N.E. (2021). Discrepancies Between Clinician and Participant Intervention Adherence Ratings Predict Percent Weight Change During a Six-Month Behavioral Weight Loss Intervention. Transl. Behav. Med..

