# Supplementary material for: Adherence of Obese Patients from Poland and Germany and Its Impact on the Effectiveness of Morbid Obesity Treatment
Source: Nutrients. 2022 Sep 19;14(18):3880. doi: 10.3390/nu14183880 (PMC9505872; doi:10.3390/nu14183880)
Supplement: Supplementary file 1 [file nutrients-14-03880-s001.zip › nutrients-1923459-supplementary.pdf]

nutrients

MDPI

Supplementary Materials

Adherence of Obese Patients from Poland and Germany and Its Impact on the Effectiveness of Morbid Obesity Treatment

Supplementary File S1. The study questionnaire.

Adherence of patients diagnosed and treated with morbid obesity (BMI ≥ 40 kg/m^2) and its impact on the effectiveness of morbid obesity treatment

We encourage patients diagnosed and treated with morbid obesity (BMI ≥ 40 kg/m^2), over the age of 18, to complete the following questionnaire "**Adherence of patients diagnosed and treated with morbid obesity (BMI ≥ 40 kg/m²)** and its impact on the effectiveness of morbid obesity treatment**". Adherence is the degree to which a patient's behavior, such as taking medications, following a diet, and making lifestyle changes, corresponds to the agreed recommendations of a health provider.**

Dear Sir or Madam,

The Department of Internal Diseases, Metabolic Disorders and Arterial Hypertension as well as the Department of Pharmacoeconomics and Social Pharmacy of the Poznan University of Medical Sciences are conducting a study regarding the association between adherence and efficacy of treatment in the population of patients diagnosed and treated with morbid obesity. The study aims to collect information necessary for scientific analysis.

The questionnaire is safe, free of charge, and anonymous. Personal data of respondents shall not be recorded. Data that could be used to identify the study participants in the future shall not be used.

Upon agreeing to participate in the study, you are asked to respond to questions in the following questionnaire, which will be used to collect the required information. We will also assess the impact of psychosocial factors on adherence and its association with the results of anti-obesity treatment. We hope that the conclusions of our study will help to organize better care for patients with obesity.

Every respondent may approach the authors to ask for information regarding the questions and to obtain a report once the analysis has been completed.

Consent to take part in the study

I have read and understood the information pertaining to the study. By responding to this questionnaire, I voluntarily consent to participate in the study and I am aware that I can withdraw my consent to participate in the subsequent parts of the study at any time without providing a reason.

1. Did you follow a slimming diet? (If not, go to question 5). Yes/No
2. If so, how many times in your life have you tried to follow a slimming diet? Please enter a number: ____
3. On whose recommendation the slimming diet was used? (Multiple answers can be selected): doctor/dietitian/Internet/ family/ friends/own motivation/ other, please enter:

4. Have you experienced the yo-yo effect? Yes/ No
5. Do you use dietary supplements to support weight loss? Yes/ No
6. If so, what kind of dietary supplements are they? Please specify:

7. Do you smoke cigarettes? (If not, go to question 9). Yes/No
8. Are you planning to quit smoking? Yes/No/Not applicable
9. Do you control the number of calories consumed per day? Yes/ No
10. In your opinion, do you eat healthily? Yes/ No
11. Do you control your blood glucose (sugar) concentration? Yes/ No
12. Do you follow the medical recommendations? Yes/ No
13. Do you play sports? (If not, go to question 18). Yes/ No
14. How many times a week do you do physical activity? 0/1/2/3/4/5/ ≥6
15. Do you go to the gym? (If not, go to question 17). Yes No
16. If so, who pays you for the gym? on its own,/to some extent it is financed by the National Health Fund/ state budget/ insurance/ not applicable
17. What kind of sport do you practice? Swimming/ running /horse riding/ roller skating/ volleyball/ cycling/dancing/ Nordic walking /walking/ football/ handball/ tennis /basketball/ other, please specify:
18. If you were allowed to attend sports classes for free, would you participate in them? Yes/No
19. Do you take any pharmacotherapy related to the treatment of obesity? Yes/ No
20. If so, what medications related to the treatment of obesity do you take? please specify:

21. Who of the qualified medical personnel conducts your obesity treatment? (Multiple answers can be selected): physician/ dietitian/ psychologist/physiotherapist/other person, please list:
22. Do you measure blood pressure yourself at home? (If not, go to question 26): Yes/No
23. If so, how often do you measure your blood pressure? Daily/twice a week/once a week/ several times a month/once a year
24. What values of systolic blood pressure predominate in your measurements?
25. What values of diastolic pressure dominate in your measurements?
26. Do you know any pro-health program related to the prevention of obesity?
27. If so, please list known pro-health programs devoted to the prevention of obesity treatment:

28. Did you take part in a pro-health program devoted to the prevention of obesity?
29. If so, please give its name:
